# The role of GLP-1 mimetics and basal insulin analogues in type 2 diabetes mellitus: guidance from studies of liraglutide

**DOI:** 10.1111/j.1463-1326.2011.01523.x

**Published:** 2012-04

**Authors:** A H Barnett

**Affiliations:** 1 BioMedical Research Centre, Heart of England NHS Foundation TrustBirmingham, UK

**Keywords:** basal insulin, GLP-1, glycaemic control, type 2 diabetes, weight loss therapy

## Abstract

In people with type 2 diabetes mellitus (T2DM), the incretin effect is reduced, but the recent advent of dipeptidyl peptidase-4 inhibitors and glucagon-like peptide (GLP)-1 agonists/analogues has enabled restoration of at least some of the function of the incretin system, with accompanying improvements in glycaemic control. Two GLP-1 receptor agonists/analogues are currently approved for the treatment of T2DM—exenatide (Byetta®, Eli Lilly & Co., Indianapolis, IN, US) and liraglutide (Victoza®, Novo Nordisk, Bagsvaerd, Denmark); a once-weekly formulation of exenatide (Bydureon®, Eli Lilly & Co.) has also been approved by the European Medicines Agency. The National Institute for Health and Clinical Excellence (NICE) has recently published guidance on the use of liraglutide in T2DM, based on evidence from the Liraglutide Effect and Action in Diabetes (LEAD) Phase III trial programme, which compared liraglutide with existing glucose-lowering therapies, such as exenatide and insulin glargine. The LEAD programme reported HbA1c reductions from 0.8 to 1.5% with liraglutide (1.2 and 1.8 mg), accompanied by low rates of hypoglycaemia and some weight loss; side effects were primarily gastrointestinal in nature (e.g. nausea and diarrhoea). Based on the findings of the LEAD studies and the NICE recommendation, liraglutide now represents an important therapy widely available in the UK for certain patient groups, including those with a body mass index (BMI) ≥35.0 kg/m^2^, and patients with a BMI <35 kg/m^2^ who are considered unsuitable for insulin and are failing to meet targets for glycaemic control with oral agents. NICE guidelines still suggest that most patients without considerable obesity (BMI <35 kg/m^2^) are probably best managed using insulin therapy. Evidence also suggests a future role for GLP-1 mimetics in combination with basal insulin.

## Introduction

Poor glycaemic control is associated with an increased risk of vascular complications in people with type 2 diabetes mellitus (T2DM) and this is, therefore, the main underlying cause of morbidity and mortality in this population [1–3]. The 10-year follow-up of patients with newly diagnosed T2DM in the UK Prospective Diabetes Study underscores the importance of achieving good glycaemic control early in the disease course. Over a median 10-year intervention, patients treated with intensive therapy had significantly lower risk of microvascular complications compared with patients receiving standard treatment [1]. A subsequent analysis of the data from 10 years after the end of the interventional period (total follow-up >20 years) also showed an additional benefit of reduced macrovascular complications and mortality with intensive treatment [4]. Thus, early intensive glycaemic control provides a ‘legacy’ of long-term morbidity and mortality reduction in patients with T2DM.

Recent years have seen a significant increase in the number of treatments available for reducing blood glucose in T2DM, including novel treatment classes with distinct efficacy and safety profiles. One such development has been the introduction of agents that reduce blood glucose via the incretin system. These include glucagon-like peptide-1 (GLP-1) agonists/analogues and dipeptidyl peptidase-4 (DPP-4) inhibitors. While an increase in treatment options is welcome, this also represents a challenge to busy physicians to make appropriate therapeutic suggestions for individual patients that must take into account patients' disease progression state, co-morbidities and concomitant treatments [5]. In response to this challenge, international and national health technology assessment and clinical advisory bodies, such as the American Diabetes Association (ADA), the European Association for the Study of Diabetes (EASD) and the National Institute for Health and Clinical Excellence (NICE) in the UK have issued guidance for physicians to assist with decision making. Most recently, NICE has published guidance on the use of liraglutide (Victoza®, Novo Nordisk, Bagsværd, Denmark), a GLP-1 analogue, which was approved by the European Medicines Agency in 2009 for use by people with T2DM.

The aim of this article is to review the current clinical evidence for liraglutide compared with other injectable therapies commonly initiated after failure of oral therapy, including other GLP-1 mimetics and basal insulin within the context of the NICE guidelines, in order to provide further guidance to physicians treating patients with T2DM who have failed on oral therapy.

## GLP-1 and the Incretin System

GLP-1 is an incretin hormone that helps maintain plasma glucose levels through regulation of insulin and glucagon [6]. Incretin hormones are secreted by gut endocrine cells (L-cells in the small and large intestine) at the beginning of a meal, and play a key role in the control of the assimilation, storage and metabolism of nutrients [7]. GLP-1 is also secreted by pancreatic islet cells and neurones in the brainstem. Incretin hormones potentiate glucose-induced insulin secretion and are responsible for around 70% of postprandial insulin secretion in healthy individuals, as well as inhibiting glucagon secretion from the pancreatic alpha-cells in the presence of hyperglycaemia, thereby reducing hepatic glucose output [6,8]. GLP-1 may also promote proliferation/neogenesis of pancreatic beta cells (animal data only) [6].

In patients with T2DM, the incretin effect is reduced, which contributes to impaired insulin regulation and glucagon secretion owing to reduced postprandial secretion of GLP-1 [9]. When exogenous GLP-1 is administered to patients, blood-glucose regulation via endogenous insulin secretion is restored [10]. GLP-1 treatment reduces overall energy intake through its actions of delaying gastric emptying and increasing satiety, and consequently may induce weight loss [11–13].

Exogenous administration of GLP-1 to regulate blood glucose is a possible therapeutic solution for T2DM; however, once subcutaneously injected, the N-terminal of the naturally occurring GLP-1 molecule is rapidly cleaved by the DPP-4 enzyme, thus generating an inactive GLP-1-(9-36) amide [14,15], resulting in a very short half-life of approximately 1.5 min [16]. As such, the frequency of exogenous GLP-1 administration required to achieve therapeutic blood-glucose regulating effects is impractical [7]. Consequently, long-acting GLP-1 receptor agonists/analogues have been developed. Currently, two GLP-1 mimetics are approved for the treatment of T2DM, exenatide (Byetta®, Eli Lilly & Co., Indianapolis, IN, US) and liraglutide, while those currently in clinical development include lixisenatide (sanofi-aventis, Paris, France), taspoglutide (Roche, Basel, Switzerland), albiglutide (GlaxoSmithKline, London, UK), LY2189265 (Eli Lilly & Co.) and CJC-1134-PC (ConjuChem, Montreal, Canada) [17,18]. A once-weekly formulation of exenatide (Bydureon®, Eli Lilly & Co.) was also approved by the European Medicines Agency in June 2011 and remains under review by the US Food and Drug Administration with a deadline of January 2012.

In targeting the incretin system, GLP-1 mimetics act principally to reduce postprandial plasma glucose (PPG) exposure, rather than fasting plasma glucose (FPG), and may have greater utility in patients with marked elevation in PPG. Much quoted evidence by Monnier et al. suggests that excursions in PPG rather than FPG are more important for patients with mild-to-moderate elevations in overall glucose control [HbA1c levels <7.3% (<56 mmol/mol)] [19]. Conversely, Monnier's data shows that the contribution of FPG levels is markedly greater than PPG in patients with very poor glycaemic control [HbA1c >10.2% (>88 mmol/mol)] [19]. Therefore, it may be inferred that treatments that preferentially target PPG, including the GLP-1 mimetics, may be more effective for patients with moderately poor glycaemic control. However, a subsequent analysis by Riddle et al. suggested that basal, rather than postprandial, blood glucose has a greater impact on patients' overall glycaemic profile and that this association is unaffected by the level of HbA1c [20]. According to Riddle et al., basal-elevated glucose levels dominate hyperglycaemic exposure in patients who have failed oral therapy, and HbA1c goals may be most successfully achieved by targeting FPG rather than PPG.

## Where do GLP-1 Mimetics Fit in the Treatment Algorithm?

In late 2010, NICE issued guidance on the use of liraglutide in T2DM [21]. These guidelines recommended that liraglutide, at a dose of 1.2 mg, can be used daily in triple-therapy regimens (in combination with metformin and a sulfonylurea, or metformin and a thiazolidinedione) in patients with T2DM, although only if used in line with previous NICE guidelines describing exenatide use [21,22]. In line with guidance for exenatide, liraglutide is recommended when control of blood glucose remains or becomes inadequate [HbA1c ≥7.5% (≥58 mmol/mol), or another higher level agreed with the individual], either in patients with a body mass index (BMI) ≥35.0 kg/m^2^ and of European descent with specific psychological or medical problems arising from high body weight, or in patients with a BMI <35 kg/m^2^ for whom insulin therapy would have significant occupational implications or in whom weight loss would benefit other significant obesity-related co-morbidities [22]. Liraglutide 1.2 mg daily is also recommended by NICE for use in dual therapy regimens (with either metformin or a sulfonylurea) in patients with T2DM who are intolerant of either metformin or a sulfonylurea, and intolerant of thiazolidinediones and DPP-4 inhibitors, or in whom these treatments are contraindicated [21].

In keeping with these stipulations, in the UK, liraglutide 1.2 mg/day now represents an additional therapy widely available for very obese (BMI ≥35.0 kg/m^2^) patients or those unsuitable for insulin who are failing to meet targets for glycaemic control with oral agents [21]. As for exenatide, NICE recommends that patients initiated on liraglutide should be monitored regularly, and that treatment should only be continued if adequate glycaemic control and weight loss are achieved (i.e. ≥1% reduction in HbA1c and ≥3% reduction in bodyweight at 6 months). Liraglutide at the higher 1.8 mg daily dose is not recommended by NICE for the treatment of T2DM on the basis that there is a lack of clinical trial evidence showing a significant benefit from increasing the dose from 1.2 to 1.8 mg, and a failure to show cost effectiveness [21].

The ADA and EASD have also updated their consensus statement to include newer medications that now have more clinical data to guide their use [23]. These outline a ranked algorithm for T2DM treatment based on validated (first line) and less well-validated (second line) therapies. The ADA/EASD treatment algorithm advocates that diabetes disease management be initiated with lifestyle changes and the use of metformin, which are both well-validated core therapies. Basal insulin or a sulfonylurea should then be added if HbA1c is ≥7% (≥53 mmol/mol) for 2–3 months despite initial intervention. This approach is favoured in patients with poor glycaemic control [HbA1c >8.5% (>69 mmol/mol)] or who have symptoms associated with hyperglycaemia. As part of second-line interventions, thiazolidinediones and the GLP-1 mimetics, liraglutide and exenatide, are advocated along with meglitinides, pramlintide and DPP-4 inhibitors because of less extensive clinical experience.

The ADA/EASD algorithm on the optimal management of hyperglycaemia has been criticized for several reasons. In particular, it has been argued that the recommended two-tier approach is not evidence-based, and that it does not offer the best quality of treatment based on the multifactorial pathophysiology of T2DM and the need for individualized therapy [24]. It should also be noted that some populations are more suited to treatment with a second-line therapy than with a first-line therapy, irrespective of how well validated each agent is, that is, a smaller evidence base does not necessarily mean that a treatment is inferior.

## Overview of Liraglutide Clinical Data

A number of long-term, Phase III, controlled, large-scale clinical trials, the Liraglutide Effect and Action in Diabetes (LEAD) trials, have investigated the effects of liraglutide compared with existing glucose-lowering therapies, either as a monotherapy or combination therapy, in patients with T2DM [25–30]. Comparator therapies in the LEAD trials include glimepiride, rosiglitazone, insulin glargine and exenatide; outcomes are summarized in Table 1. The clinical data for liraglutide, including the LEAD data, have been extensively reviewed previously [31].

**Table 1:** Summary of the liraglutide effect and action in diabetes (LEAD) clinical programme in type 2 diabetes.

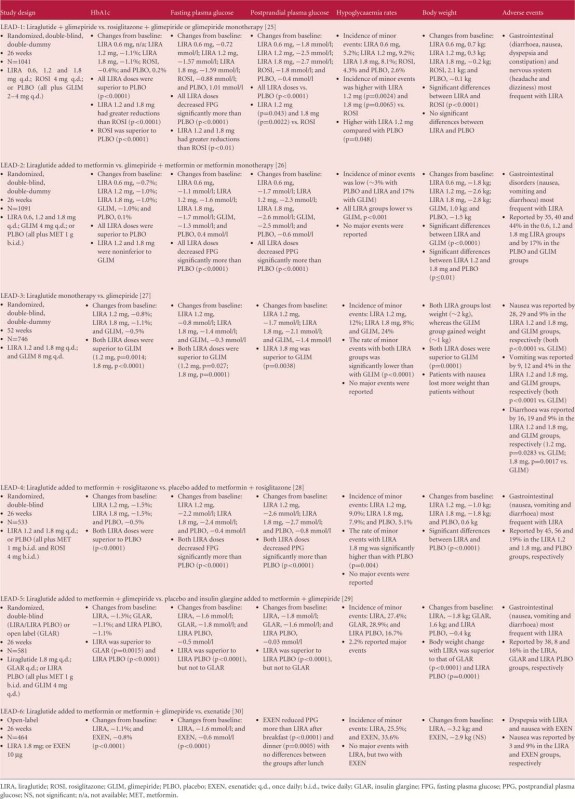

### Liraglutide Clinical Efficacy Data

Overall, in the LEAD studies, reductions in HbA1c of 0.8– 1.5% were observed with liraglutide therapy at doses of 1.2 and 1.8 mg (Table 1), with more patients achieving glycaemic targets compared with those receiving glimepiride (with or without metformin) in the LEAD-2 and -3 studies [26,27]. The LEAD-2 trial enrolled patients who had previously received either mono- or dual oral therapy, and liraglutide appeared to have a greater propensity to reduce blood glucose levels in patients who had previously been treated with monotherapy rather than dual therapy, possibly because of the former group having less advanced disease and greater *β*-cell function [26].

In the LEAD-6 trial, liraglutide was directly compared with the other available GLP-1 mimetic, exenatide, in people with T2DM inadequately controlled on maximally tolerated doses of metformin, sulfonylurea or both oral agents. Patients received liraglutide 1.8 mg/day or exenatide 10 µg twice daily over 26 weeks of treatment [30]. Significantly greater improvements in HbA1c were achieved in the liraglutide-treated patients compared with those who received exenatide (estimated treatment difference −0.33; 95% confidence intervals (CI) −0.47, −0.18; p < 0.0001), and more patients in the liraglutide group achieved HbA1c <7% (≥53 mmol/mol) than in the exenatide group. The reduction in FPG was significantly greater with liraglutide compared with exenatide (estimated treatment difference −1.01 mmol/l; 95% CI −1.37, −0.65; p<0.0001) but PPG control was less effective after breakfast and dinner with liraglutide vs. exenatide. LEAD-6 used the higher liraglutide dose of 1.8 mg/day, rather than the lower dose of 1.2 mg/day recommended by NICE [21]. Weight loss was comparable between the groups (liraglutide −3.24 kg vs. exenatide −2.87 kg).

**Figure 1:**
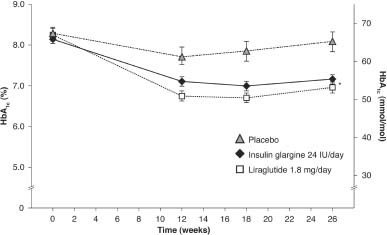
LEAD-5 trial: mean HbA1c change (%) from baseline over time (last observation carried forward, intention-to-treat population). *p<0.05 for liraglutide vs. insulin glargine and placebo. LEAD, liraglutide effect and action in diabetes. Reprinted with permission from Springer [29].

**Figure 2:**
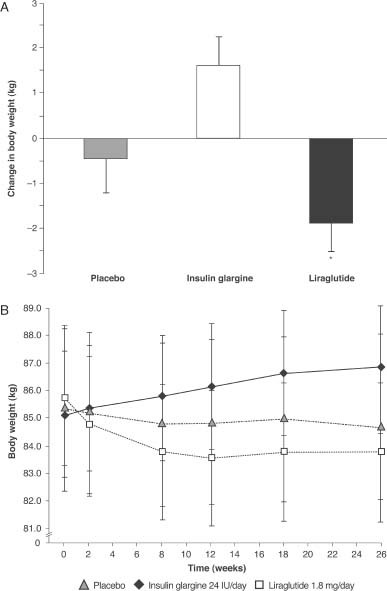
LEAD-5 trial: change in (A) body weight over time, and (B) body weight from baseline [mean (SD)]. Data are last observation carried forward, intent-to-treat population; *Liraglutide vs. insulin glargine (p<0.0001) and placebo (p=0.0001). SD, standard deviation; LEAD, liraglutide effect and action in diabetes. Reprinted with permission from Springer [29].

The LEAD-5 study was a direct head-to-head trial comparing liraglutide with insulin glargine over 26 weeks [29]. Liraglutide was again administered at the higher dose of 1.8 mg/day, while insulin glargine was titrated using the AT.LANTUS algorithm, designed for ‘ease of initiation’, to yield a mean insulin glargine dose of 24 IU. A third ‘liraglutide placebo’ arm was included in the LEAD-5 study to provide a control. Liraglutide reduced HbA1c significantly more than insulin glargine (1.33 vs. 1.09%, respectively; p=0.0015; figure 1); both liraglutide and insulin glargine reduced HbA1c significantly compared with placebo (p<0.0001) [29]. FPG and PPG were also improved in both active treatment groups (Table 1). Patients receiving liraglutide had a mean weight change from baseline of −1.8 kg, compared with −0.4 kg (p=0.0001) for those receiving placebo and +1.6 kg (p<0.0001) for those receiving insulin glargine (figure 2) [29]. As in the LEAD-6 trial, the liraglutide dose of 1.8 mg/day was higher than that recommended in the NICE guidelines while, given the diabetes population used in this study, the mean insulin glargine dose of 24 IU was considered low by the NICE evidence review group, given the mean dose of insulin glargine administered in other published studies. This low insulin glargine dose may be attributable to the fact that insulin glargine was titrated using the AT.LANTUS algorithm rather than the more rigorous Treat-to-Target algorithm, which shows greater efficacy in HbA1c reduction.

**Figure 3:**
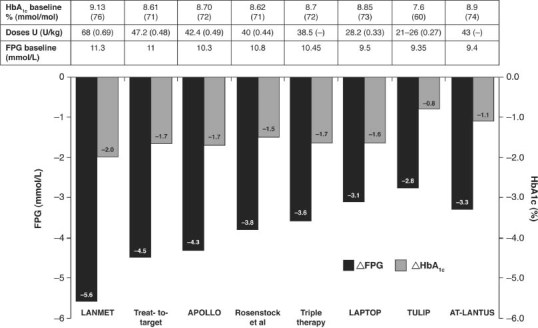
A strong relationship is evident between HbA1c and FPG reductions in clinical trials with insulin glargine [29,32–39].

The study outcomes of LEAD-5 also suggest under-titration of insulin glargine in this trial. Comparison of the FPG levels achieved here (Table 1) with other similar trials shows that markedly greater reductions in FPG with insulin glargine have been achieved in trials that utilized the Treat-to-Target algorithm [32], suggesting that greater reductions in HbA1c might have occurred with more appropriate insulin glargine titration. Indeed several trials have shown that reductions in FPG with insulin glargine are strongly correlated with reductions in HbA1c (figure 3) [32–39].

## Liraglutide Clinical Safety Data Including Incidence of Hypoglycaemia

The most frequent adverse events (AEs) observed with liraglutide in the LEAD trials were gastrointestinal (GI) symptoms, particularly nausea and diarrhoea [31]. At the dose recommended by NICE (1.2 mg/day), rates of nausea ranged from 10.5 to 27.5%. In the LEAD-5 study, which utilized the higher 1.8 mg/day liraglutide dose, nausea and diarrhoea occurred in 13.9 and 10.0% of liraglutide-treated patients, respectively, compared with 1.3 and 1.3% of insulin glargine-treated patients [29]. Withdrawal from the LEAD-5 trial because of AEs was greater in the liraglutide vs. the insulin glargine group (4.7 vs. 2.1%) [29]. In the LEAD-6 trial comparing 1.8 mg/day liraglutide with 10 µg exenatide twice daily, tolerability was comparable between the two groups, although nausea was less persistent with liraglutide (estimated treatment rate ratio 0.448, p<0.0001). In addition to the GI symptoms, concerns regarding acute pancreatitis and altered renal function have been reported with GLP-1 mimetics [7,40].

Across the liraglutide Phase III trial programme, the incidence of hypoglycaemia associated with liraglutide varied from 0.03 to 1.9 events per patient [31]. In LEAD-6, a direct comparison of liraglutide and exenatide showed that minor hypoglycaemia was less frequent with liraglutide than with exenatide (1.93 vs. 2.60 events per patient per year; rate ratio 0.55; 95% CI 0.34, 0.88; p=0.0131). No major hypoglycaemic events were reported in the LEAD-2, -3 or -6 studies [31]. In LEAD-1, one patient receiving liraglutide plus glimepiride experienced a major hypoglycaemic event, while liraglutide-treated patients experienced more minor hypoglycaemic events than those who received rosiglitazone treatment [25]. In the LEAD-5 study, five (2.2%) liraglutide-treated patients reported major hypoglycaemic events compared with none in the insulin glargine group [29]. The proportion of patients experiencing minor hypoglycaemia was similar in both the liraglutide and insulin glargine groups (27.4 vs. 28.9%).

### Use of Liraglutide in Special Populations—Obesity

**Figure 4:**
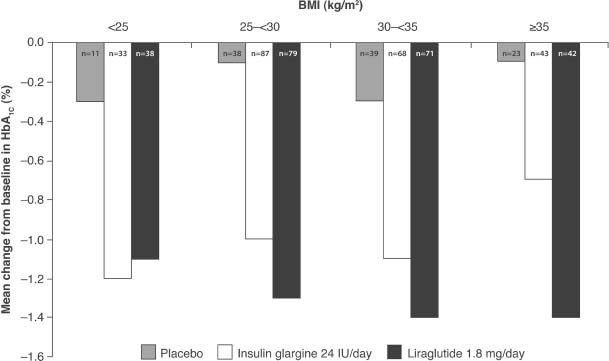
LEAD-5 trial: Mean change from baseline in HbA1c (%) at Week 26 by baseline body mass index category [41]. Subgroup analysis of the intent-to-treat, last observation carried forward population. LEAD, liraglutide effect and action in diabetes.

A recent analysis of the efficacy of liraglutide was conducted by the US Food and Drug Administration based on the results of five key studies from the LEAD programme [25–29], which identified that between 43 and 74% of patients enrolled were obese (BMI ≥30 kg/m^2^) [41]. Across the liraglutide arms of the five studies, weight loss occurred in most patients, although some actually gained weight [41]. The greatest weight loss occurred in patients with an initial BMI ≥35 kg/m^2^. In the LEAD-5 trial, the mean HbA1c change from baseline in the liraglutide arm was also greater in patients with a higher mean BMI, while this relationship was not evident with insulin glargine (figure 4) [41].

## Combining Basal Insulin Analogues with GLP-1 Mimetics

Evidence suggests that both basal insulin analogues and GLP-1 mimetics play a key role in achieving glycaemic control in patients with T2DM. Basal insulin analogues are a highly effective method of reducing fasting and overall blood glucose, while GLP-1 mimetics are advocated for those where weight gain is a concern [23]. The progressive nature of T2DM means that many individuals require multiple therapeutic strategies to maintain glycaemic targets. Traditionally, glycaemic control using insulin regimens has been achieved with basal insulin to target FPG, followed by the addition of bolus insulin to cover PPG excursions, if required. However, very intensive insulin regimens are associated with a higher risk of hypoglycaemia and weight gain, which can be a burden on patients [42–44]. The combination of basal insulin with a GLP-1 mimetic is a potential solution to this problem for some patients with T2DM. As GLP-1 analogues target PPG excursions, they could, in theory, complement the activity of fasting control with basal insulins.

Combining a GLP-1 mimetic with basal insulin may provide improvements in glycaemic control together with better weight management and low rates of hypoglycaemia, potentially increasing patient treatment satisfaction. However, there may also be an increased incidence of GI side effects in some patients. A number of studies have been reported for a basal insulin/GLP-1 mimetic combination, the majority of which were conducted with exenatide and insulin glargine [45–48]. These studies show that the combination regimen does indeed provide improvements in HbA1c and PPG along with weight loss and no substantial increase in the risk of hypoglycaemia [45–48]. Similar findings have been noted in preliminary reports of clinical experience with liraglutide plus insulin [49–51], and a preliminary report of a randomized study showed that the addition of insulin detemir to metformin and liraglutide (1.8 mg/day) in patients with T2DM generated a substantial reduction in HbA1c, with a small reduction in bodyweight and low rates of hypoglycaemia [52]. The GLP-1 mimetic lixisenatide, which currently remains in development, has been specifically investigated for use in combination with insulin glargine in two Phase III studies: GetGoal-L and GetGoal-L Asia. Data from GetGoal-L Asia has recently been made available and showed that adding once-daily lixisenatide for patients insufficiently controlled on basal insulin, with or without a sulfonylurea, significantly improved HbA1c compared with placebo, with particularly pronounced reductions in PPG levels [53]. The GetGoal-L studies were performed with a ‘free combination’, that is, the two components were administered separately as two injections, but both lixisenatide and insulin glargine are administered once daily and a formulation combining both agents into a single pen device for once-daily injection is planned.

While the basal insulin/GLP-1 mimetic combination is an attractive proposition, further investigation is required. Future studies should assess the relative benefits of the GLP-1 mimetics in combination with basal insulin. Head-to-head comparison of liraglutide and exenatide in the absence of basal insulin in the LEAD-6 study showed that exenatide had a significantly greater impact on PPG levels than liraglutide, while liraglutide preferentially targeted FPG levels (Table 1). In theory, the greater PPG effects of exenatide over liraglutide may be preferential in combination with the FPG-targeting effects of basal insulin. Preliminary data for the GetGoal-X head-to-head study of exenatide and lixisenatide showed comparable HbA1c- and FPG-reducing effects for these agents, but did not report their impact on PPG levels [54].

## Conclusions

There are long-term data to support the importance of early achievement and maintenance of tight glycaemic control to reduce the risk of T2DM-related complications. However, the attainment of these targets is hindered by the complex pathophysiology of T2DM and the drawbacks associated with currently available therapies, such as risk of hypoglycaemia and potential weight gain. Hence, there is a need to tailor diabetes therapy according to individual needs and the level of glycaemic control required.

GLP-1 analogues, such as liraglutide, represent an important new therapeutic option in diabetes management and the recent NICE recommendations for liraglutide facilitate its use in the individualization of treatment in certain patient groups. These include T2DM patients who cannot adequately control their diabetes with oral therapy, and who have considerable obesity, or where hypoglycaemia and/or weight gain is particularly problematic or for whom therapy with insulin would have significant occupational implications. Key attributes of the GLP-1 mimetics are low risk of hypoglycaemia and, compared with sulfonylureas, thiazolidinediones and insulin, they are not associated with significant weight gain. However, it should be noted that in the head-to-head trial of liraglutide and insulin glargine, rates of overall hypoglycaemia were comparable, although liraglutide was shown to provide a small but statistically significant greater improvement in HbA1c [29]. Interpretation of the relative efficacy and safety seen with liraglutide and insulin glargine in the LEAD-5 study should take into account doses of these agents, which according to NICE recommendations was high for liraglutide (1.8 mg/day) and lower than expected for insulin glargine (24 IU).

GLP-1 mimetics may be associated with GI side effects that are not seen with insulin use, and the impact of these AEs in terms of patient burden as well as treatment adherence and persistence can often be underestimated by physicians.

When initiating an injectable therapy, such as basal insulin or a GLP-1 mimetic, physicians should be mindful of the relative benefits of the individual therapies with respect to improvements in glycaemic control and weight management, particularly in more difficult to treat patient groups. For example, patients with particularly poor glycaemic control may benefit more from the potentially greater efficacy of insulin therapy. Evidence supports a strong correlation between reductions in FPG and HbA1c with insulin glargine, but a weaker association between these two variables with liraglutide. Insulin glargine or indeed another basal insulin may be a better choice when adopting a ‘fix the fasting first' approach to treatment [55]. However, the balance between the need for glycaemic control and weight management is likely to favour treatment with GLP-1 mimetics for patients with high BMIs. The relative safety profiles of both treatment types should also be carefully considered; for example, balancing the benefits of a potential for reduced hypoglycaemia with GLP-1 mimetics against the higher incidence of GI AEs. The use of GLP-1 mimetics as an add-on to basal insulin looks to be extremely promising and the FDA approved the use of exenatide in combination with insulin glargine in October 2011. In conclusion, GLP-1 mimetics are a welcome addition to the diabetes treatment armamentarium.
